# Deep Reinforcement Learning-Driven Jamming-Enhanced Secure Unmanned Aerial Vehicle Communications

**DOI:** 10.3390/s24227328

**Published:** 2024-11-16

**Authors:** Zhifang Xing, Yunhui Qin, Changhao Du, Wenzhang Wang, Zhongshan Zhang

**Affiliations:** 1 School of Information and Electronics, Beijing Institute of Technology, Beijing 100081, China; 3220195138@bit.edu.cn; 2 National School of Elite Engineering, University of Science and Technology Beijing, Beijing 100081, China; qinyunhui@ustb.edu.cn; 3 School of Cyberspace Science and Technology, Beijing Institute of Technology, Beijing 100081, China; 3120215682@bit.com (W.W.); zhangzs@bit.edu.cn (Z.Z.)

**Keywords:** unmanned aerial vehicle (UAV), jamming UAV, deep reinforcement learning, sequential decision problem

## Abstract

Despite its flexibility, unmanned aerial vehicle (UAV) communications are susceptible to eavesdropping due to the open nature of wireless channels and the broadcasting nature of wireless signals. This paper studies secure UAV communications and proposes a method to optimize the minimum secrecy rate of the system by using interference technology to enhance it. To this end, the system not only deploys multiple UAV base stations (BSs) to provide services to legitimate users but also assigns dedicated UAV jammers to send interference signals to active or potential eavesdroppers to disrupt their eavesdropping effectiveness. Based on this configuration, we formulate the optimization process of parameters such as the user association variables, UAV trajectory, and output power as a sequential decision-making problem and use the single-agent soft actor-critic (SAC) algorithm and twin delayed deep deterministic policy gradient (TD3) algorithm to achieve joint optimization of the core parameters. In addition, for specific scenarios, we also use the multi-agent soft actor-critic (MASAC) algorithm to solve the joint optimization problem mentioned above. The numerical results show that the normalized average secrecy rate of the MASAC algorithm increased by more than 6.6% and 14.2% compared with that of the SAC and TD3 algorithms, respectively.

## 1. Introduction

Unmanned aerial vehicles (UAVs) have been widely adopted in emergency rescue, instant messaging, and other fields due to their excellent flexibility and cost-effectiveness. Flexibly deployed UAVs can carry communication payloads and act as airborne base stations (BSs) to provide access services for ground users.

However, the open nature of wireless channels and the broadcasting nature of wireless signals significantly increase the security risks of UAV communication. Malicious users can eavesdrop on the communication content of legitimate users by intercepting and stealing UAV signals, which results in significant security threats, especially in applications involving sensitive data transmissions, such as surveillance, disaster response, and secure communication networks [1,2,3]. Therefore, ensuring the communication security of legitimate users and effectively combating potential eavesdroppers has become a major challenge in the field of UAV communication. In order to address the above challenges, a new method called “friendly UAV jamming” has been proposed, which sends special interference signals to eavesdroppers to prevent them from obtaining eavesdropped content, thereby ensuring the information security of legitimate users [4,5].

Unfortunately, operating the aforementioned UAV security communication mechanism requires solving complex mathematical problems that traditional methods are powerless to address. Therefore, researchers have begun to use new methods based on artificial intelligence to solve these problems. Notable achievements include deep reinforcement learning (DRL)-based methods [6,7], including the twin delayed deep deterministic policy gradient (TD3) algorithm [8], proximal policy optimization (PPO) [9], and the soft actor-critic (SAC) algorithm [10]. Additionally, the multi-agent DRL approach is also effective in providing distributed and online solutions. For instance, the authors of [11] introduced multi-agent DRL approaches to jointly optimize critical parameters, such as UAV trajectories, user association variables, and transmit power, in multi-UAV-assisted communication systems.

Ensuring secure communication involves strategies to protect data transmissions between UAVs and legitimate users from eavesdropping, which becomes especially challenging in open environments. To solve the UAV security communication problem, many relevant studies exist [6,12,13]. UAVs are often deployed to monitor or communicate within designated areas, ensuring consistent coverage over time for tasks like surveillance, data collection, or relaying communication signals. Periodic coverage ensures that UAVs revisit specific areas regularly, which is essential in dynamic environments.

Therefore, this paper studies the periodic coverage-assisted secure communication of UAVs with coverage evaluation constraints. Unlike existing studies [14,15], this paper fully considers scenarios with active and potential eavesdroppers. Specifically, we use multiple UAV BSs to provide services to legitimate users while also deploying a certain number of UAV jammers to send interference signals to eavesdroppers. Considering the limited carrying capacity of UAVs, as described in [16], this paper adopts a cyclic coverage estimation scheme to improve the service capability of UAV clusters.

The main purpose of this paper is to propose a new strategy to help legitimate users maximize their minimum secrecy rate. The optimization objective presents a complex mixed-integer nonlinear optimization problem. This problem is mathematically intractable because maximizing the minimum secrecy rate requires the simultaneous optimization of the user association variables, UAV trajectory, and output power. However, due to the complex nature of the coverage constraints, the mobility of the UAVs, and the discrete nature of the user association variables, problem solving becomes highly challenging. DRL algorithms may be more suitable for addressing these problems. Therefore, this paper formulates the optimization objective as a sequential decision-making problem. The DRL method (i.e., single-agent SAC and TD3 algorithms) and the multi-agent SAC (MASAC) algorithm can effectively solve these problems. The numerical results show that the MASAC algorithm is superior in accumulating discounted rewards but at the cost of higher time complexity during the training process. In contrast, the SAC algorithm performs best in terms of stability and can obtain better cumulative discounted rewards than the TD3 algorithm.

The rest of this paper is organized as follows. Section 2 describes the system model and problem formulation. Deep reinforcement learning-based solutions for joint optimization are discussed in Section 3. The numerical results are provided in Section 4, and Section 5 concludes this paper.

*Notations:* ${\left(\cdot \right)}^{T}$ represents the transpose, and |·| and ∥·∥ refer to the modulus and Euclidean norm, respectively. ${\left[\cdot \right]}^{+}$ means that the calculation result inside the square brackets is non-negative. E(·) denotes the mathematical expectation. ∪ and ≫ represent the union and “much greater than” operations, respectively.

## 2. System Model and Problem Formulation

**Figure 1:**
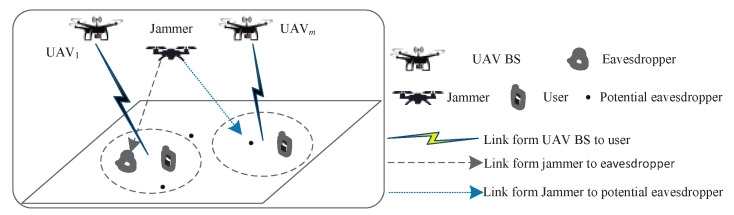
The jamming-enhanced secure UAV communication deployment in the target area.

This paper mainly studies the secure communication model of jamming-enhanced UAVs. Figure 1 shows the deployment of a jamming-enhanced secure UAV communication system. Assume that the number of single-antenna users is *U*, and these users are served by *M* UAV BSs. In addition, assume that the system is equipped with *J* UAV jammers, which protect legitimate user information security by sending noise-like interference signals to eavesdroppers. Without loss of generality, assume that the number of ground eavesdroppers is *I*. Furthermore, the sets of legitimate users, eavesdroppers, UAV BSs, and UAV jammers are denoted by U={1,2,⋯U}, I={1,2,⋯I}, M={1,2,⋯M}, and J={1,2,⋯J}, respectively. In addition to the *I* deterministic eavesdroppers, this paper also considers the possibility of potential eavesdroppers snooping on legitimate information.

Assume that *K* potential eavesdroppers are randomly distributed within the target area, where $s_{k}={\left[c_{k},d_{k}\right]}^{T}\in s$, with k∈K={1,⋯K} signifying the position of the *k*-th latent eavesdropper.

To facilitate both system trajectory planning and resource allocation, we preset the flight cycle of the UAV as *T*, which can be divided into *N* time slots and has a duration of $\delta_{T}=T/N$, where N∈1,⋯N. As long as each time slot is short enough and the UAV’s flight speed is moderate, we assume that its position remains almost unchanged during this period. The flight altitude, or hovering altitude, of each UAV is expressed as *H*. In addition, $\Theta_{m}\left[n\right]={\left[X_{m}\left[n\right],Y_{m}\left[n\right]\right]}^{T}$ and $\theta_{j}\left[n\right]={\left[x_{j}\left[n\right],y_{j}\left[n\right]\right]}^{T}$ are used to characterize the *m*-th UAV BS and the *j*-th UAV jammer in the *n*-th time slot, respectively. For simplicity, the horizontal positions of all legitimate and eavesdropping UAVs are represented as $\Theta \left[n\right]=\left\{X_{1}\left[n\right],\cdots X_{M}\left[n\right];Y_{1}\left[n\right],\cdots Y_{M}\left[n\right]\right\}$ and $\theta \left[n\right]=\left\{x_{1}\left[n\right],\cdots x_{J}\left[n\right];y_{1}\left[n\right],\cdots y_{J}\left[n\right]\right\}$, respectively. In addition, the position of the legitimate user *u* in time slot *n* is denoted by $A_{u}\left[n\right]={\left[a_{u}\left[n\right],b_{u}\left[n\right]\right]}^{T}$ with zero altitude. We also use $B_{i}\left[n\right]={\left[a_{i}^{\prime }\left[n\right],b_{i}^{\prime }\left[n\right]\right]}^{T}$ to denote the location of the *i*-th deterministic eavesdropper.

Considering the limited carrying capacity of UAVs, this paper proposes a periodic coverage evaluation mechanism for UAV interference and potential eavesdropping. This mechanism ensures that the system achieves strong anti-eavesdropping capabilities at the lowest energy cost. Specifically, we assume that at least one potential eavesdropping coverage state needs to be calculated within each frame period. For ease of analysis, the total number of frames is set to $L=T/T_{L}$, where $T_{L}$ represents the frame length. Next, we assume that the number of time slots contained in each coverage frame is $N_{L}=N/L$.

Once the UAV jammer *j* intends to access the potential eavesdropper $s_{k}$ in time slot *n*, we set $c_{j,k}\left[n\right]=0$. Otherwise, $c_{j,k}\left[n\right]=1$. Meanwhile, we limit access to a maximum of one potential eavesdropper per time slot. This separation coverage evaluation mechanism effectively reduces computational overhead. Based on the above analysis, the associated variables of potential eavesdroppers must meet the following conditions:

(1) $$\begin{array}{cc} \sum_{n=\left(l-1\right)N_{L}+1}^{lN_{L}}c_{j,k}\left[n\right] & =1,\forall j,k; \end{array}$$

(2) $$\begin{array}{cc} \sum_{k=1}^{K}c_{j,k}\left[n\right] & \leq 1,\forall j,n; \end{array}$$

(3) $$\begin{array}{cc} \sum_{j=1}^{J}c_{j,k}\left[n\right] & \leq 1,\forall k,n; \end{array}$$

(4) $$\begin{array}{cc} c_{j,k}\left[n\right] & \in \{0,1\}. \end{array}$$

In the following, we employ $C_{s_{k}}\in \left\{0,1\right\}$ to denote the coverage state at $s_{k}$ in time slot *n*, as given by

(5) $$C_{s_{k}}\left[n\right]=\left\{\begin{array}{c} 1,\;\gamma_{s_{k}}\left[n\right]\geq \mu ;\\ 0,\;\mathrm{otherwise}, \end{array}\right.$$

where $\gamma_{s_{k}}$ denotes the signal-to-noise-plus-interference ratio (SINR) of $s_{k}$. This equation indicates that as long as $\gamma_{s_{k}}$ is not lower than the system preset threshold μ, $s_{k}$ will be considered as staying inside the coverage range.

Like in [17], we also use the reference signal receiving power (denoted by RP) to determine the value of the SINR, as given by

(6) $$\begin{array}{cc} \gamma_{s_{k}}\left[n\right] & =\frac{\mathrm{RP}(s_{k})}{\sum_{j=1}^{J}q_{j,k}\left[n\right]g_{j,k}\left[n\right]-\mathrm{RP}\left(s_{k}\right)+\sigma^{2}}\\ \; & =\frac{\max(q_{j,k}\left[n\right]g_{j,k}\left[n\right])}{\sum_{j=1}^{J}q_{j,k}\left[n\right]g_{j,k}\left[n\right]-\max\left(q_{j,k}\left[n\right]g_{j,k}\left[n\right]\right)+\sigma^{2}}. \end{array}$$

where $q_{j,k}$ represents the transmission power of the *j*-th UAV jammer, $g_{j,k}$ denotes the power gain of the jammer, and  $\sigma^{2}$ stands for the variance of additive white Gaussian noise (AWGN).

The channel power gain from the *j*-th jammer to $s_{k}$ is

(7) $$\begin{array}{cc} g_{j,k}\left[n\right]= & g_{0}d^{-2}\left(\theta_{j}\left[n\right],s_{k}\right)\\ = & \frac{g_{0}}{H^{2}+{∥\theta_{j}\left[n\right]-s_{k}\left[n\right]∥}^{2}}, \end{array}$$

where $g_{0}$ represents the channel power at a reference distance of 1 m.

Let $\alpha_{m,u}\left[n\right]$ represent the association coefficient between the *m*-th UAV BS and the *u*-th legitimate user, where $\alpha_{m,u}\left[n\right]=1$ implies that the legitimate user *u* in time slot *n* is served by the *m*-th UAV; otherwise, $\alpha_{m,u}\left[n\right]=0$. Assume that each UAV has the ability to serve multiple targets simultaneously, while each target is exclusively served by only one UAV, i.e., $\alpha_{m,u}\left[n\right]\in \left\{0,1\right\},\forall m,u$.

(8) $$\sum_{m=1}^{M}\alpha_{m,u}\left[n\right]=1,\forall u\in U,$$

Considering the limited spectrum resources used by the system, UAVs adopt the principle of spectrum reuse to increase system capacity while ensuring that the interference they receive can be controlled at an acceptable level.

As described in [18], the data rate of the *u*-th legitimate user is given by

(9) $$R_{m,u}\left[n\right]=\alpha_{m,u}\left[n\right]\log\left(1+\gamma_{m,u}\left[n\right]\right),$$

where

(10) $$\gamma_{m,u}\left[n\right]=\frac{p_{m,u}\left[n\right]g_{m,u}\left[n\right]}{\sum_{m^{\prime }\in M\setminus m}p_{m^{\prime },u}\left[n\right]g_{m^{\prime },u}\left[n\right]+\sigma^{2}}$$

denotes the user’s SINR, $p_{m,u}$ represents the transmit power of the UAV *m*, and $\sigma^{2}$ stands for the noise variance at the receiver. The power gain of the *u*-th legitimate user is thus given by

(11) $$\begin{array}{cc} g_{m,u}\left[n\right] & =g_{0}d^{-2}\left(\Theta_{m}\left[n\right],A_{u}\left[n\right]\right)\\ \; & =\frac{g_{0}}{H^{2}+{∥\Theta_{m}\left[n\right]-A_{u}\left[n\right]∥}^{2}}. \end{array}$$

Similarly, the data rate of eavesdropping is expressed as

(12) $$R_{E,i}^{m,u}\left[n\right]=\log\left(1+\gamma_{E,i}^{m,u}\left[n\right]\right),$$

where

(13) $$\gamma_{E,i}^{m,u}\left[n\right]=\frac{p_{m,u}\left[n\right]g_{m,i}\left[n\right]}{I},$$

$I=\sum_{m^{\prime }\in M\setminus m}p_{m^{\prime },u}\left[n\right]g_{m^{\prime },i}\left[n\right]+\sum_{j=1}^{J}q_{j,i}\left[n\right]g_{j,i}\left[n\right]+\sigma^{2}$, $q_{j,i}$ represents the transmit power of the *i*-th UAV jammer, and the power gains are $g_{m,i}\left[n\right]=g_{0}d^{-2}\left(\Theta_{m}\left[n\right],B_{i}\left[n\right]\right)=\frac{g_{0}}{H^{2}+{∥\Theta_{m}\left[n\right]-B_{i}\left[n\right]∥}^{2}}$ and $g_{j,i}\left[n\right]=g_{0}d^{-2}\left(\theta_{j}\left[n\right],B_{i}\left[n\right]\right)=\frac{g_{0}}{H^{2}+{∥\theta_{j}\left[n\right]-B_{i}\left[n\right]∥}^{2}}$.

Following (9) and (12), the worst achievable average secrecy rate of the *u*-th legitimate user over a *T*-duration in the presence of eavesdroppers can be given by

(14) $$R_{\sec}^{u}=\frac{1}{N}\sum_{n=1}^{N}\sum_{m=1}^{M}{\left[R_{m,u}\left[n\right]-\max_{\forall i}R_{E,i}^{m,u}\left[n\right]\right]}^{+},$$

where ${\left[x\right]}^{+}=\max\left\{x,0\right\}$.

In the following, we focus on maximizing the minimum secrecy rate by optimizing the parameters, including trajectory planning, user association variables, and power distribution of UAVs. The optimization goal can thus be formulated as

(15) $$\left(P0\right)\;\max_{\alpha ,\Theta ,\theta ,p,q}\min R_{\sec}^{u}.$$

(15a) $$\begin{array}{cc} \; & \left(1),(2),(3),(4),(8\right) \end{array}$$

(15b) $$\begin{array}{cc} \; & c_{j,k}\left[n\right]\gamma_{s_{k}}\left[n\right]\geq c_{j,k}\left[n\right]\mu ,\;\forall j,k \end{array}$$

(15c) $$\begin{array}{cc} \; & 0\leq p_{m,u}\left[n\right]\leq p_{\max},\;\forall u\in U \end{array}$$

(15d) $$\begin{array}{cc} \; & 0\leq q_{j,i}\left[n\right]\leq p_{\max},\;\forall j\in J,i\in I \end{array}$$

(15e) $$\begin{array}{cc} \; & 0\leq q_{j,k}\left[n\right]\leq p_{\max},\;\forall j\in J,k\in K, \end{array}$$

where α denotes the user association variables; p and q represent the transmission power of the UAV BS and UAV jammer, respectively; and Θ and θ are the coordinates of the UAVs. For a given coverage-evaluating frequency, the coverage constraints for $s_{k}$ are shown in (15b), and the power constraints are shown in (15c)–(15e).

It is evident that the optimization objective (15) involves a mixed-integer nonlinear non-convex problem, making it difficult to solve using traditional iterative optimization methods. Therefore, we transform the above optimization problem into a sequential decision-making problem and adopt a DRL-based approach to achieve the joint optimization of the user association variables, power allocation, and trajectory planning in jamming-enhanced secure UAV communication systems.

## 3. Deep Reinforcement Learning-Based Solutions for Joint Optimization

The nonlinearity and non-convexity of the optimization objective (15) pose significant mathematical challenges for solving the aforementioned joint optimization problem. Considering that trajectory planning, user association variables, and power allocation are all sequential decision problems, the above optimization process can be reconstructed as a Markov decision process (MDP). This section investigates single-agent and multi-agent DRL solutions.

### 3.1. The Single-Agent DRL Solution

Let (S,A,R,γ) represent the tuple of the MDP, where *S* denotes the state space, *A* corresponds to the action space, and *R* signifies the reward function. The long-term cumulative discounted reward can be expressed as $R\left(\pi \right)=E\left[\sum_{t=1}^{T}\gamma^{t-1}\left[R\left(s_{t},a_{t},s_{t+1}\right)\right]\right]$, where γ∈[0,1) represents the discount factor. The constituent elements are defined as follows:

- State space *S*: $s_{t}\in S$ represents the state during time slot *t*, encompassing the coordinates of the UAV BSs, the UAV jammers, and the legitimate users:
  (16) $$\begin{array}{c} s_{t}=\left\{X_{t},Y_{t},U_{x,t},U_{y,t},I_{x,t},I_{y,t},K_{x,t},K_{y,t}\right\}, \end{array}$$
  where $\left(X_{t},Y_{t}\right)$ denotes the coordinates of all UAVs in time slot *t*, which is composed of the coordinates of UAV BSs $\left(M_{x,t},M_{y,t}\right)$ and the coordinates of UAV jammers $\left(J_{x,t},J_{y,t}\right)$. $\left(U_{x,t},U_{y,t}\right)$ represents the coordinates of legitimate users. $\left(I_{x,t},I_{y,t}\right)$ and $\left(K_{x,t},K_{y,t}\right)$ are the coordinates of the active and potential eavesdroppers, respectively.
- Action space *A*: $a_{t}\in A$ represents the action taken during time slot *t*, encompassing the user association variables, the allocation of power to both legitimate users and eavesdroppers, and the variations in UAV locations:
  (17) $$a_{t}=\left\{\alpha_{t},p_{t},q_{t},\Delta X_{t},\Delta Y_{t}\right\},$$
  where $p_{t}$ and $q_{t}$ denote the allocated power by communication UAVs and jamming UAVs, respectively, within time slot *t*. The flight displacement of UAVs is represented by $\left(\Delta X_{t},\Delta Y_{t}\right)$, which is composed of $\left(\Delta M_{x,t},\Delta M_{y,t}\right)$ and $\left(\Delta J_{x,t},\Delta J_{y,t}\right)$.
- Reward function: The reward function $r_{t}$, $r_{t}\in R$ comprises two components, i.e., the secrecy rate and the penalty of the coverage evaluation. The coverage evaluation of UAV jammers is denoted by $R_{c}$, where $R_{c}=c_{j,k}\left[t\right]\gamma_{s_{k}}\left[t\right]-c_{j,k}\left[t\right]\mu$. Therefore, the reward function can be expressed as
  (18) $$r_{t}=\min_{u\in U}R_{\sec}^{u}+\beta R_{c},$$
  where β denotes the penalty factor.

Single-agent DRL algorithms, such as the SAC algorithm and TD3 algorithm, can be used to solve this problem. Take the SAC algorithm as an example. The SAC algorithm aims to maximize the long-term cumulative discounted reward while maximizing the strategy entropy, as given by $\max E\sum_{t=1}^{T}\gamma^{t-1}[r_{t}\left(s_{t},a_{t}\right)-\rho \log\pi_{\phi }\left(a_{t}\left|s_{t}\right)\right]$. Here, ρ and $\pi_{\phi }$ represent the temperature parameter and actor network with parameter ϕ, respectively.

In the SAC algorithm framework, there exist two main critic networks, i.e., $Q_{\theta_{1}}$ and $Q_{\theta_{2}}$, with network parameter vectors $\theta_{1}$ and $\theta_{2}$. There also exist two target critic networks, i.e., $Q_{\theta_{1}^{\prime }}$ and $Q_{\theta_{2}^{\prime }}$, with parameter vectors $\theta_{1}^{\prime }$ and $\theta_{2}^{\prime }$. The purpose of the critic networks is to fit the soft Q-function of the agent. Furthermore, the stochastic actor network $\pi_{\phi }$ generates actions based on the state of the agent.

**Figure 2:**
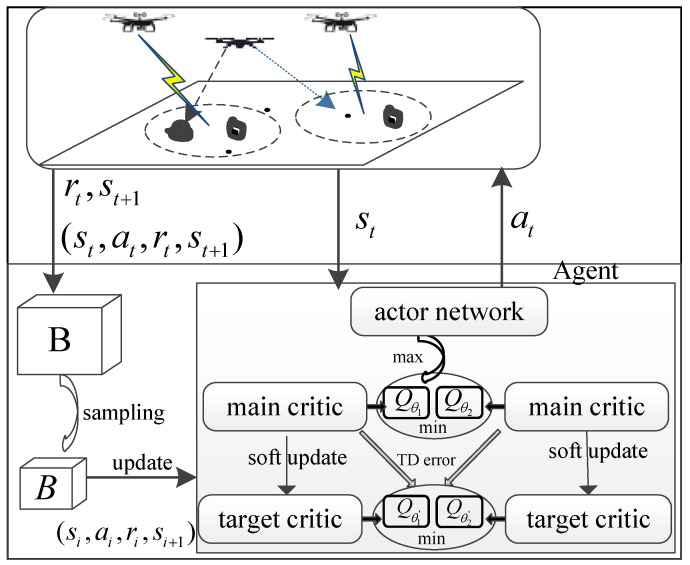
Diagram of the single-agent SAC algorithm for the jamming-enhanced secure UAV communication network.

Figure 2 shows a diagram of the single-agent SAC algorithm for the jamming-enhanced secure UAV communication network. In each time slot, the agent interacts with the environment to generate a new experience tuple $\left(s_{t},a_{t},r_{t},s_{t+1}\right)$, which is then stored in the replay memory buffer B. As time passes, the number of tuples in the replay buffer gradually increases until a sufficient number of samples are reached. In order to optimize the parameter vectors of the critic and actor networks, the system randomly samples a batch of tuples *B* to form the replay buffer B, that is, ${\left(s_{i},a_{i},r_{i},s_{i+1}\right)}_{i=1}^{\left|B\right|}$.

The critic network can be updated by minimizing

(19) $$J\left(\theta_{i}\right)=\frac{1}{\left|B\right|}\sum {\left[\left(Q_{\theta_{i}}\left(s_{t},a_{1},\cdots a_{t}\right)-y_{t}\right)\right]}^{2},$$

where $s_{t},a_{t}\in B$, i=1,2, and $y_{t}$ denotes the target value of the main critic network in time slot *t*, which is given by

(20) $$\begin{array}{c} y_{t}=r_{t}+\gamma \left(\min_{i=1,2}Q_{\theta_{i}^{\prime }}\left(s_{t+1},a_{t+1}\right)\right.\\ \left.-\rho \log_{\pi_{\phi }}\left({\tilde{a}}_{t+1}|s_{t+1}\right)\right),{\tilde{a}}_{t+1}=\pi_{\phi }\left(\cdot |s_{t+1}\right). \end{array}$$

The actor network can be updated according to

(21) $$\begin{array}{c} J\left(\phi \right)=\frac{1}{\left|B\right|}\sum [\min_{i=1,2}Q_{\theta_{i}}\left(s_{t},a_{t}\right)\\ -\rho \log\pi_{\phi }(a_{t}\left|s_{t}\right)]. \end{array}$$

In addition, the temperature parameter ρ can be updated according to [10], and the target critic networks follow the soft update rule $\theta_{i}^{\prime }\leftarrow \epsilon \theta_{i}+\left(1-\epsilon \right)\theta_{i}^{\prime },i=1,2$, where ϵ is the soft update parameter.

**Figure 3:**
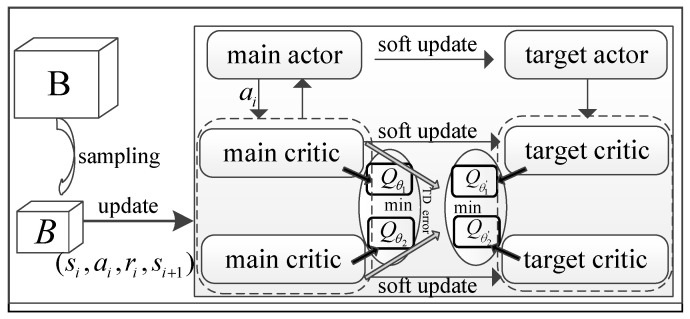
Diagram of the agent in the single-agent TD3 algorithm.

Note that the TD3 algorithm utilized in this paper is also a typical single-agent DRL solution and is similar to the SAC algorithm, with an off-policy actor-critic mechanism. The actor and critic networks are depicted in Figure 3, and their parameter updating follows existing studies [8,19]. For simplicity, the details of the TD3 algorithm are not elaborated further.

### 3.2. The Multi-Agent DRL Solution

In the multi-agent DRL solution, each UAV BS represents an agent. We use (O,A,R,γ) to denote the tuple of the MDP, where *O* represents the global observation of all agents. The main elements are explained as follows:

- Observation space *O*: $o_{m,t}\in O$ represents the state of agent *m* during time slot *t*, ∀m∈M∪J. The local observation space $o_{m,t}$ mainly consists of the coordinates of UAVs, the coordinates of legitimate users, and those of the active and potential eavesdroppers:
  (22) $$\begin{array}{cc} o_{m,t}= & \{X_{x_{m},t},Y_{y_{m},t},U_{x_{m},t},U_{y_{m},t},\\ & I_{x_{m},t},I_{y_{m},t},K_{x_{m},t},K_{y_{m},t}\}, \end{array}$$
  where $X\in M_{x,t}\cup J_{x,t}$ and $Y\in M_{y,t}\cup J_{y,t}$.
- Action space *A*: $a_{m,t}\in A$ represents the action of agent *m* during time slot *t*, and it is composed of the user associative variables, the allocation of power to both legitimate users and eavesdroppers, and the variations in UAV locations:
  (23) $$\begin{array}{cc} a_{m,t}= & \{\alpha_{m,t},p_{m,t},q_{m,t},\Delta M_{x_{m},t},\Delta M_{y_{m},t},\\ & \Delta J_{x_{m},t},\Delta J_{y_{m},t}\}. \end{array}$$
- Reward function *R*: The reward function $r_{m,t}$, $r_{m,t}\in R$ for the agent *m* comprises both the secrecy rate and the penalty of the coverage evaluation. The coverage evaluation of UAV jammers is denoted by $R_{c}=c_{j,k}\left[t\right]\gamma_{s_{k}}\left[t\right]-c_{j,k}\left[t\right]\mu$. The reward function can be written as
  (24) $$r_{m,t}=\min_{u\in U}R_{\sec}^{u}+\beta R_{c}.$$

In this paper, we employ the MASAC algorithm to solve the problem, where each agent corresponds to a UAV and comprises two main critic networks (i.e., $Q_{\theta_{m,1}}$, $Q_{\theta_{m,2}}$), two target critic networks (i.e., $Q_{\theta_{m,1}^{\prime }}$, $Q_{\theta_{m,2}^{\prime }}$, ∀m∈M∪J), and one actor network $\pi_{\phi_{m}}$.

In the training process, the agent *m* is designed to maximize

$$E\left\{\sum_{t=1}^{T}\gamma^{t-1}[r_{m,t}\left(o_{m,t},a_{m,t}\right)-\rho_{m}\log\pi_{\phi_{m}}\left(a_{m,t}\left|o_{m,t}\right)\right]\right\}.$$

**Figure 4:**
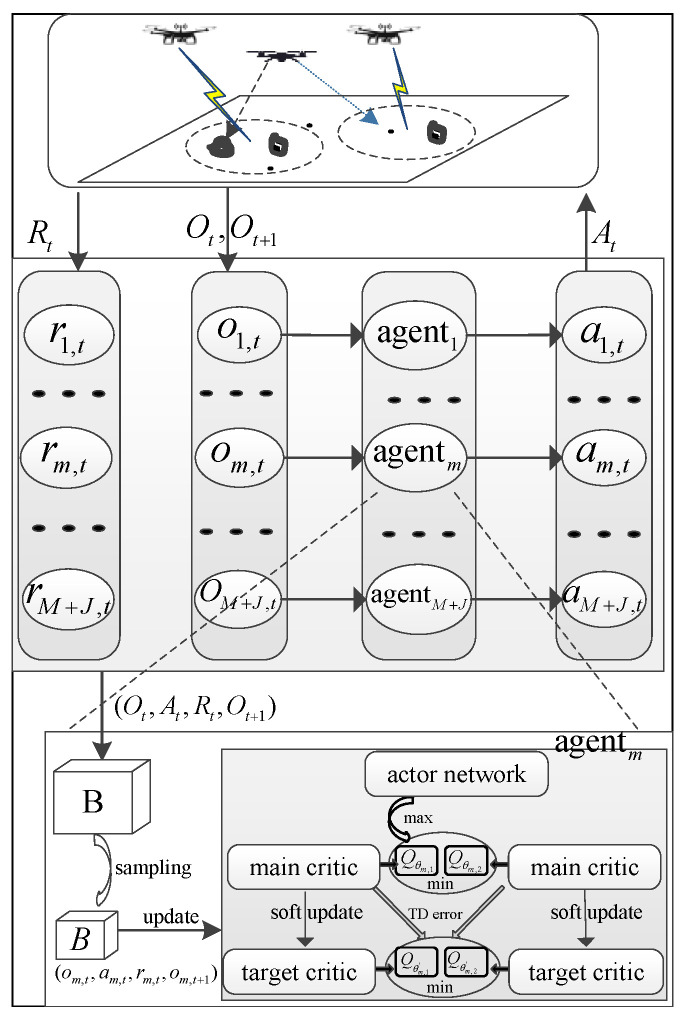
Diagram of the MASAC algorithm for the jamming-enhanced secure UAV communication network.

Figure 4 shows a diagram of the MASAC algorithm for this jamming-enhanced secure UAV communication network. After each interaction with the environment, the experience tuple $\left(O_{t},A_{t},R_{t},O_{t+1}\right)$ is gradually generated and stored in *B*. To update the neural network parameters, a minibatch of experience tuples *B* is randomly sampled from B.

The MASAC algorithm follows the centralized training and decentralized execution mechanism. The critic network can be updated by minimizing the soft Bellman residuals:

(25) $$J\left(\theta_{m,i}\right)=\frac{1}{\left|B\right|}\sum {\left[\left(Q_{\theta_{m,i}}\left(O_{t},a_{1,t},\cdots a_{m,t}\right)-y_{m,t}\right)\right]}^{2},$$

where $O_{t},a_{m,t}\in B$, i=1,2, and $y_{m,t}$ is the target value of the main critic network in time slot *t*, as given by

(26) $$y_{m,t}=\frac{1}{\left|B\right|}\sum \left[r_{m,t}+\gamma V\left(Q_{t+1}\right)\right],$$

where $V\left(Q_{t+1}\right)=\min_{i=1,2}Q_{\theta_{m,i}^{\prime }}\left(O_{t+1},\cdots {\tilde{a}}_{m,t+1}\right)-\rho_{m}\log\pi_{\phi_{m}}\left({\tilde{a}}_{m,t+1}|o_{m,t+1}\right)$ and ${\tilde{a}}_{m,t+1}∼\pi \left(\cdot |o_{m,t+1}\right)$.

The actor network can be updated according to

(27) $$\begin{array}{c} J\left(\phi_{m}\right)=\frac{1}{\left|B\right|}\sum [\min_{i=1,2}Q_{\theta_{m,i}}\left(O_{t},a_{1,t},\cdots a_{m,t}\right)\\ -\rho_{m}\log\pi_{\phi_{m}}\left(a_{m,t}|o_{m,t}\right)]. \end{array}$$

In addition, the target critic networks of each agent follow the soft update rule $\theta_{m,i}^{\prime }\leftarrow \epsilon \theta_{i}+\left(1-\epsilon \right)\theta_{m,i}^{\prime },i=1,2$, where ϵ is the soft update parameter, and the temperature parameter $\rho_{m}$ can be updated according to [20].

The pseudocode of the MASAC is presented in Algorithm 1.
**Algorithm 1** MASAC algorithm for jamming-enhanced secure UAV communications  1:For each ${\mathrm{UAV}}_{m}$, initial main network parameters $\theta_{m,i}$, $\phi_{m}$, set target network parameters $\theta_{m,i}^{\prime }$, ←$\theta_{m,i}$, i=1,2, ∀m∈M∪J.  2:**for** each episode **do**  3:    Initial the global observation $O_{t}$  4:    **for** t←1,T **do**  5:          **for** m←1,M∪J **do**  6:               Select policy $a_{m,t}∼\pi_{\phi_{m}}\left(\cdot \right)$  7:          **end for**  8:          Execute actions $A_{t}=\left(a_{m,t},\cdots a_{m,t}\right)$  9:          Observe reward $R_{t}$ and the next global observation $O_{t+1}$10:          Store the tuple $\left(O_{t},A_{t},R_{t},O_{t+1}\right)$ in B11:          $O_{t}\leftarrow O_{t+1}$12:          **for** m←1,M∪J **do**13:               Sample minibatch *B* from B14:               Update the main critic network parameter:15:               $\theta_{m,i}\leftarrow \nabla_{\theta_{m,i}}J\left(\theta_{m,i}\right),i=1,2$16:               Update actor network parameter:17:               $\phi_{m}\leftarrow \nabla_{\phi_{m}}J\left(\phi_{m}\right)$18:               Update target critic network $\theta_{m,i}^{\prime },i=1,2$ following the soft update rule19:               Update temperature parameter $\rho_{m}$ according to [20]20:          **end for**21:      **end for**22:**end for**

### 3.3. Computational Complexity Analysis

In this section, we investigate the optimization of the secrecy rate based on single-agent and multi-agent DRL methods and analyze their complexity. The complexity of these algorithms is determined by the neural network framework of the algorithms. In our DRL solution, both the critic and actor networks are four-layer fully connected networks, with an architecture consisting of one input layer, two hidden layers, and one output layer. Let $n_{a_{1}}$ and $n_{a_{2}}$ represent the number of nodes in hidden layer 1 and hidden layer 2 in the actor network, respectively. Meanwhile, let $n_{c_{1}}$ and $n_{c_{2}}$ represent the number of nodes in hidden layer 1 and hidden layer 2 in the critic network.

First, we analyze the complexity of the single-agent SAC algorithm. It can be deduced that both the state space and the action space have a dimension of 2(M+J+U+I+K) and 2MU+JI+JK+2(M+J), respectively, corresponding to the number of input nodes $n_{i}$ and output nodes $n_{o}$ of the actor network, i.e., $n_{i}=2\left(M+J+U+I+K\right)$ and $n_{o}=2MU+JI+JK+2\left(M+J\right)$. According to [21], the time complexity at each training step of each actor network is given by

(28) $$\begin{array}{c} O_{a}=O\left({n_{i}}^{2}+n_{a_{1}}^{2}+n_{a_{2}}^{2}+{n_{o}}^{2}\right). \end{array}$$

Similarly, the time complexity of each critic network at each step can be calculated as

(29) $$\begin{array}{c} O_{c}=O\left({\left(n_{i}+n_{o}\right)}^{2}+n_{c_{1}}^{2}+n_{c_{2}}^{2}\right). \end{array}$$

Considering all the main and target networks of the SAC algorithm at each step, the time complexity in the training process is $O_{\mathrm{train}}^{\mathrm{SAC}}=O_{a}+4O_{c}$.

During the testing process, only the actor network is used to determine the action interacting with the environment, and its time complexity depends only on the matrix multiplication complexity of the layers. The calculation can be expressed as

(30) $$\begin{array}{c} O_{\mathrm{test}}^{\mathrm{SAC}}=O\left(n_{i}*n_{a_{1}}+n_{a_{1}}*n_{a_{2}}+n_{a_{2}}*n_{o}\right). \end{array}$$

Note that there are six neural networks in the TD3 algorithm, including two main critic networks, two target critic networks, one main actor network, and one target actor network. The time complexity at each step in the training process can be described as $O_{\mathrm{train}}^{\mathrm{TD}3}=2O_{a}+4O_{c}$. During the testing process, as we only applied the optimal actor network to decide the actions, the time complexity of each step is approximately equivalent to that of the SAC algorithm.

For the MASAC algorithm, we can deduce that the local observation space has a dimension of 2(1+J+U+I+K), denoted by $n_{m,i}$, ∀m∈M∪J, and the action space has a dimension of 2MU+JI+JK+2(1+J), denoted by $n_{m,o}$, ∀m∈M∪J. Therefore, the time complexity for the *m*-th agent at each training step of each actor network is given by $O_{m,a}=O\left({\left(n_{m,i}\right)}^{2}+n_{a_{1}}^{2}+n_{a_{2}}^{2}+{\left(n_{m,o}\right)}^{2}\right)$. Similarly, the time complexity of each critic network at each step is $O_{m,c}=O\left({\left(n_{m,i}+n_{m,o}\right)}^{2}+n_{c_{1}}^{2}+n_{c_{2}}^{2}\right)$.

Therefore, the time complexity for all agents in each training step is

(31) $$O_{\mathrm{train}}^{\mathrm{MASAC}}=\sum_{m=1}^{M+J}O_{m,a}+4\sum_{m=1}^{M+J}O_{m,c}.$$

In the testing process, the time complexity of all agents can be calculated as

(32) $$O_{\mathrm{test}}^{\mathrm{MASAC}}=\sum_{m=1}^{M+J}O\left(n_{m,i}*n_{a_{1}}+n_{a_{1}}*n_{a_{2}}+n_{a_{2}}*n_{m,o}\right).$$

In summary, the number of input nodes for an actor network is usually smaller than the number of input nodes for a critic network, implying that the testing process has comparatively lower computational complexity, whereas the training process has much higher computational complexity. Table 1 shows the computational complexity of different algorithms in the training and testing processes. Moreover, it can also be concluded that $\sum_{m=1}^{M+J}O_{m,a}+4\sum_{m=1}^{M+J}O_{m,c}\gg O_{a}+4O_{c}$; therefore, the MASAC algorithm has higher time complexity than the SAC algorithm in the training process, that is, $O_{\mathrm{train}}^{\mathrm{MASAC}}\gg O_{\mathrm{train}}^{\mathrm{SAC}}$.

## 4. Numerical Results

The simulation environment was set up in a square area with a side length of 1 km. Assume that legitimate users were randomly distributed throughout the entire area, and the positions of UAVs in the target area were randomly initialized at the beginning of the simulation. We considered a periodic coverage-assisted area comprising three UAV BSs, two UAV jammers, 20 legitimate users, two ground eavesdroppers, and five latent eavesdroppers. The flight period was set to T=50 s, the coverage evaluation frame length was $T_{L}=20$ s, the predetermined flight altitude of the drones was 150 m, and each UAV was associated with the nearest legitimate users. Moreover, the time slot length was set to $\delta_{t}=0.25$ s, and the threshold for the coverage evaluation was set to μ=−3 dB. The reference channel power was $g_{0}=-60$ dB [22].

The experiments were simulated using Python v3.7, and the deep learning framework used was PyTorch. Both the critic and the policy networks were implemented as four-layer fully connected networks, with 128 neurons implemented in each hidden layer. Each episode comprised 50 time slots. Furthermore, the discount factor γ and the number of experience tuples |B| were set to 0.96 and 256, respectively, while the learning rates of the critic and actor networks were set to 0.0001 and 0.00001, respectively.

**Figure 5:**
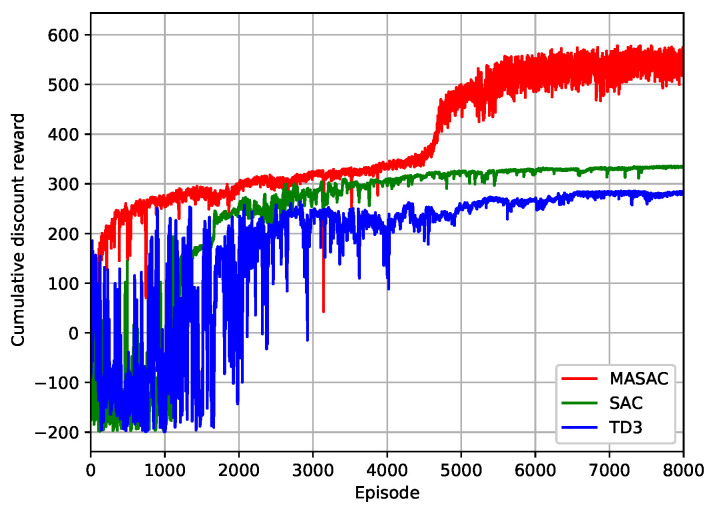
The cumulative discounted reward versus the training episodes.

Figure 5 shows the cumulative discounted return of the DRL algorithms versus the training episodes. It can be seen that the MASAC algorithm performed best in terms of convergence speed and cumulative discounted return compared to the other algorithms. The SAC algorithm demonstrated the best stability during the training phase and better cumulative discounted return performance than the TD3 algorithm. This is because the multi-agents in the MASAC algorithm have a better ability to explore and cooperate. On one hand, multiple agents explore different parts of the environment simultaneously, which helps the agents learn better policies compared to a single agent. On the other hand, multiple agents cooperate in their actions to achieve shared or individual goals more efficiently, and each agent can specialize in a role or a subset of tasks, leading to better performance. However, the MASAC algorithm had higher time complexity than the other algorithms, that is, $O_{\mathrm{train}}^{\mathrm{MASAC}}\gg O_{\mathrm{train}}^{\mathrm{SAC}}$. The MASAC algorithm was quite time-consuming, which can be attributed to its centralized training and decentralized execution mechanism.

**Figure 6:**
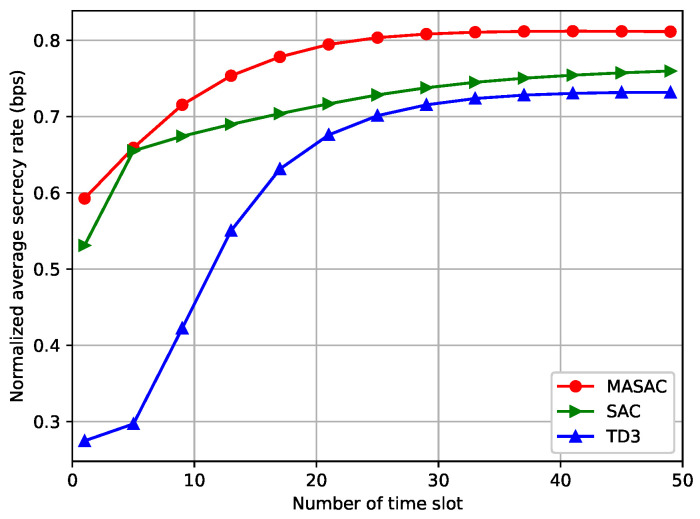
The normalized average secrecy rate versus the number of time slots.

To verify the effectiveness of the DRL-based solutions, we saved the neural network parameters after each algorithm’s training was completed. Then, only the actor network was utilized to determine the action interacting with the environment and further calculate the corresponding secrecy rate in the testing process. Figure 6 shows the normalized average secrecy rate versus the number of time slots. It can be observed that the secrecy rate for each algorithm increased as the number of time slots increased, with the secrecy rate for the MASAC algorithm increasing by more than 6.6% and 14.2% compared to that of the SAC and TD3 algorithms, respectively. The simulation results reveal the validity of the DRL algorithms in finding the effective user association variables, UAV trajectory, and power allocation policy for the considered scenarios.

**Figure 7:**
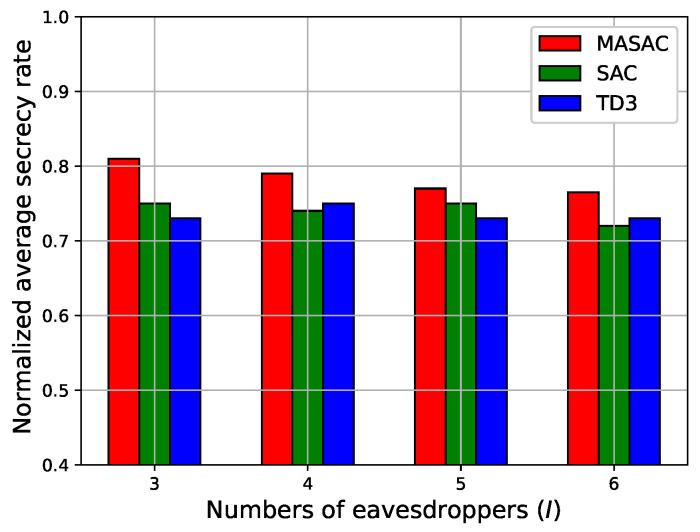
The normalized average secrecy rate versus the number of ground eavesdroppers.

**Figure 8:**
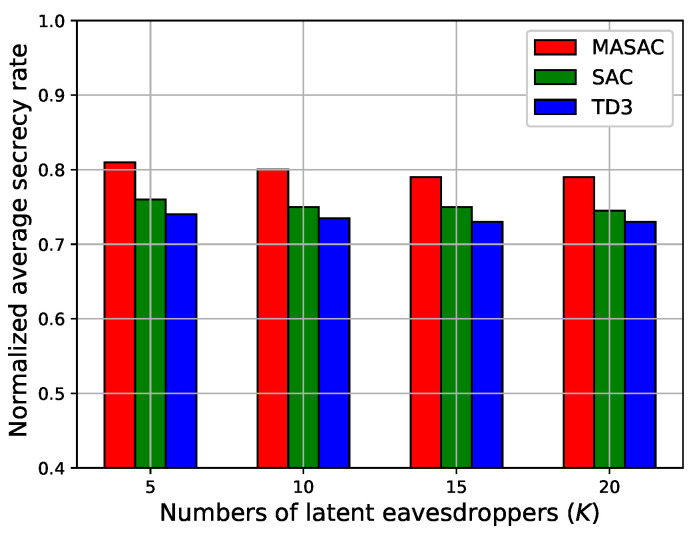
The normalized average secrecy rate versus the number of latent eavesdroppers.

Finally, we studied the relationship between the normalized average secrecy rate and the number of eavesdroppers, as shown in Figure 7 and Figure 8. These experiments included three UAV BSs, two UAV jammers, and 20 legitimate users. In the solutions, we saved the parameters of their respective actor networks after each algorithm’s training was completed, then loaded them to decide on the variables in the testing process, and finally calculated the corresponding secrecy rate. It can be observed in Figure 7 that the secrecy rate tended to decrease as the number of eavesdroppers increased. The MASAC algorithm achieved the best secrecy rate compared with the other algorithms. In Figure 8, it can be seen that the number of latent eavesdroppers had less influence on the average secrecy rate. It can be deduced that the existing UAV jammers effectively ensured the secrecy rate in this scenario. Moreover, the MASAC algorithm achieved the best secrecy rate in terms of different numbers of latent eavesdroppers.

## 5. Conclusions

This paper investigated the problem of maximizing the minimum achievable secrecy rate in interference-enhanced UAV secure communication systems, with a focus on addressing challenges such as joint user association variables, power allocation, and UAV trajectory optimization. Due to the high complexity of solving the optimization objective function, we transformed the problem into a Markov decision process with low complexity and used both single-agent DRL and multi-agent DRL algorithms (including SAC, TD3, and MASAC) to solve these problems. The simulation results show that the MASAC algorithm is effective in accumulating discounted rewards but at the cost of higher time complexity during the training process. In contrast, the SAC algorithm performs the best in terms of stability, and its cumulative discounted reward is better than that of the TD3 algorithm.

In future work, we will explore secure transmission for integrated sensing and communication (ISAC)-enabled UAV networks. These single-agent and multi-agent DRL algorithms will be exploited to solve the user association variables, UAV trajectory planning, and power allocation problems in UAV-ISAC networks.

## Figures and Tables

**Table 1:** Computational complexity for different DRL algorithms in our considered scenarios.

Algorithm	Training Process	Testing Process
MASAC	$\sum_{m=1}^{M+J}O_{m,a}+4\sum_{m=1}^{M+J}O_{m,c}$	$\sum_{m=1}^{M+J}O\left(n_{m,i}*n_{a_{1}}+n_{a_{1}}*n_{a_{2}}+n_{a_{2}}*n_{m,o}\right)$
SAC	$O_{a}+4O_{c}$	$O(n_{i}*n_{a_{1}}+n_{a_{1}}*n_{a_{2}}+n_{a_{2}}*n_{o})$
TD3	$2O_{a}+4O_{c}$	$O(n_{i}*n_{a_{1}}+n_{a_{1}}*n_{a_{2}}+n_{a_{2}}*n_{o})$

## Data Availability

The raw data supporting the conclusions of this article will be made available by the authors on request.
